# A two-component system regulates gene expression of the type IX secretion component proteins via an ECF sigma factor

**DOI:** 10.1038/srep23288

**Published:** 2016-03-21

**Authors:** Tomoko Kadowaki, Hideharu Yukitake, Mariko Naito, Keiko Sato, Yuichiro Kikuchi, Yoshio Kondo, Mikio Shoji, Koji Nakayama

**Affiliations:** 1 Division of Microbiology and Oral Infection, Department of Molecular Microbiology and Immunology, Graduate School of Biomedical Sciences, Nagasaki University, Nagasaki 852-8588, Japan; 2 Division of Frontier Life Science, Department of Medical and Dental Sciences, Graduate School of Biomedical Sciences, Nagasaki University, Nagasaki 852-8588, Japan; 3 Department of Microbiology, Tokyo Dental College, Tokyo 101-0061, Japan; 4 Department of Pediatric Dentistry, Graduate School of Biomedical Sciences, Nagasaki University, Nagasaki 852-8588, Japan

## Abstract

The periodontopathogen *Porphyromonas gingivalis* secretes potent pathogenic proteases, gingipains, via the type IX secretion system (T9SS). This system comprises at least 11 components; however, the regulatory mechanism of their expression has not yet been elucidated. Here, we found that the PorY (PGN_2001)-PorX (PGN_1019)-SigP (PGN_0274) cascade is involved in the regulation of T9SS. Surface plasmon resonance (SPR) analysis revealed a direct interaction between a recombinant PorY (rPorY) and a recombinant PorX (rPorX). rPorY autophosphorylated and transferred a phosphoryl group to rPorX in the presence of Mn^2+^. These results demonstrate that PorX and PorY act as a response regulator and a histidine kinase, respectively, of a two component system (TCS), although they are separately encoded on the chromosome. T9SS component-encoding genes were down-regulated in a mutant deficient in a putative extracytoplasmic function (ECF) sigma factor, PGN_0274 (SigP), similar to the *porX* mutant. Electrophoretic gel shift assays showed that rSigP bound to the putative promoter regions of T9SS component-encoding genes. The SigP protein was lacking in the *porX* mutant. Co-immunoprecipitation and SPR analysis revealed the direct interaction between SigP and PorX. Together, these results indicate that the PorXY TCS regulates T9SS-mediated protein secretion via the SigP ECF sigma factor.

Periodontal disease is a major cause of tooth loss^1^,^2^ and is one of the most common infectious diseases in humans^3^, resulting in the destruction of periodontal tissue and alveolar bone loss^4^. The main periodontopathogen is a Gram-negative anaerobic bacterium, *Porphyromonas gingivalis*^5^, which secretes highly catalytic proteases known as gingipains that disrupt host defence mechanisms^6^,^7^. Gingipains consist of Arg-specific cysteine proteinases (Rgp) encoded by *rgpA* and *rgpB*, and the Lys-specific cysteine proteinase (Kgp) encoded by *kgp*^8^,^9^,^10^. Gingipains have signal peptides at their N-terminus which enable them to pass through the inner membrane in association with Sec machinery.

Comparative genome analysis revealed that gingipains are transported across the outer membrane by the novel Por secretion system (PorSS)^11^. The proteins constituting PorSS differ from those of other known secretion systems; however, they are encoded by the genomes of many bacteria in the phylum *Bacteroidetes*^12^. Therefore, PorSS is also known as the type IX secretion system (T9SS), which involves at least 11 component proteins: PorK (PGN_1676), PorL (PGN_1675), PorM (PGN_1674), PorN (PGN_1673), PorP (PGN_1677), PorQ (PGN_0645), PorT (PGN_778), PorU (PGN_0022), PorV (PGN_0023), PorW (PGN_1877), and Sov (PGN_0832). Mutations in *porX* (encoding PGN_1019) and *porY* (encoding PGN_2001) previously showed a deficiency in the secretion of gingipains. Amino acid sequence similarities indicate that PorX and PorY are response regulator (RR) and sensor histidine kinase (HK) components, respectively, of a two component system (TCS)^11^.

TCS is one of the most common signal transduction mechanisms in bacteria to sense and respond to environmental stresses. In many pathogens, the system is also known to modulate the transcription of genes associated with multiple pathogenic events such as invasion into host cells, biofilm formation, chemotaxis, lipopolysaccharide modification, and resistance to antibiotics^13^,^14^,^15^,^16^. TCS typically consists of two protein components: an HK, which autophosphorylates in response to environmental stimuli, and an RR, which receives the phosphate group from HK and controls the regulation of target gene expression.

Tiling DNA microarray analysis using a custom tiling DNA array chip with the genome sequence of *P. gingivalis* ATCC 33277 previously revealed that 20 genes were down-regulated in the *porX* deletion mutant to less than 60% of the wild-type parental strain^11^. These 20 genes included *porT*, *sov*, *porK*, *porL*, *porM*, *porN*, and *porP* of T9SS. Reverse transcriptase (RT)-PCR analysis confirmed that these genes were down-regulated in the *porY* mutant, suggesting that PorX and PorY have roles in regulating the expression of T9SS component-encoding genes. Usually, HK- and RR-encoding genes are located tandemly, or nearby on a chromosome. However, *porX* and *porY* are ‘orphan’ genes because they are located separately on the *P. gingivalis* chromosome^17^.

To mediate adaptation processes in response to environmental signals, bacteria possess multiple signalling pathways. Extracytoplasmic function (ECF) sigma factors are involved in one of the major bacterial signalling pathways as well as TCS^18^. ECF sigma factors, which belong to the sigma-70 family of proteins, facilitate the alteration of gene expression by binding to and guiding the core RNA polymerase. Six ECF sigma factors, PGN_0274, PGN_0319, PGN_0450, PGN_0970, PGN_1108, and PGN_1740, are encoded by the *P. gingivalis* ATCC 33277 genome. Previous studies have indicated that PGN_0274 and PGN_0450 in strain ATCC 33277 (PG0162 and PG1660 in strain W83, respectively) are involved in the expression of gingipains^19^,^20^, that PGN_1740 (SigH, PG1827 in W83) is required for the adaptation to oxygen stress^21^, that PGN_1108 (PG1318 in W83) has a role in the regulation of mutation frequency^22^, and that PGN_0274 and PGN_1740 are involved in biofilm formation^23^.

In this study, we examined whether PorX and PorY function as a cognate pair of the TCS. We also investigated the relationship of the ECF sigma factor PGN_0274, which we designated SigP (sigma factor for expression of *por* genes), with T9SS in association with the TCS.

## Results

### Subcellular localization of PorX and PorY in *P. gingivalis*

**Figure 1:**
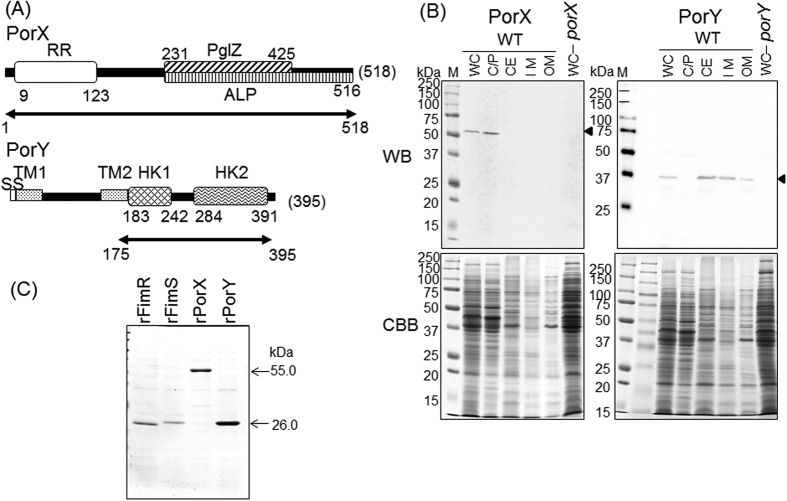
Structure and localization of PorX and PorY. (**A**) Schematic representation of the functional domains of PorX and PorY. The symbols denote the following, RR, response regulatory domain; PglZ, PglZ motif; ALP, alkaline phosphatase-like core domain; SS, signal sequence; TM, transmembrane domain; HK1, histidine kinase phosphoacceptor domain; HK2, histidine kinase ATPase domain. Arrows below the schema indicate the recombinant proteins generated in this study. The domains were predicted by the Kyoto Encyclopedia of Genes and Genomes (KEGG) Sequence Similarity DataBase (KEGG SSDB). (**B**) Subcellular localization of PorX and PorY in *P. gingivalis.* Fractionated cell lysates of the wild-type strain ATCC 33277 were subjected to immunodetection with antisera against PorX and PorY. WC, whole cell lysate; C/P, cytoplasm/periplasm; CE, cell envelope; IM, inner membrane; OM, outer membrane. Arrowheads indicate the immunoreacting PorX and PorY bands. (**C**) SDS-PAGE profile of the purified recombinant proteins.

The predicted amino acid sequences and expected functional domains of PorX and PorY are shown in Fig. 1A. PorX has a putative RR receiver domain at the N-terminus, which is predicted to receive a phosphoryl group from its cognate HK partner in the TCS. At the C-terminal region of this RR domain is a PglZ domain, which is a member of the alkaline phosphatase clan. PorY is predicted to have two transmembrane domains following to a typical signal peptide at the N-terminus. Two HK-associated sequences are located in the C-terminal half of PorY: a phosphoacceptor domain (HK1) and an ATPase domain (HK2).

Localization of these proteins was determined by subcellular fractionation and Western blot analysis (Fig. 1B). A 55-kDa protein band immunoreactive to the anti-PorX antibody was detected in the cytoplasmic/periplasmic fraction of the wild-type protein, and was absent from the whole cell lysate of a *porX* mutant. Additionally, a 37-kDa protein reactive to the anti-PorY antibody was mainly detected in the inner membrane fraction and partially in the outer membrane fraction, and was absent from the whole cell lysate of a *porY* mutant. Thus, PorX appears to localize in the cytoplasm/periplasm and PorY appears to span the inner membrane, which are the typical locations of RRs and HKs. The computational prediction with TMHMM (http://www.cbs.dtu.dk/services/TMHMM-2.0/) and DAS (http://www.sbc.su.se/~miklos/DAS/) also suggest that PorY includes two helical lipid-interfacing domains, which is preferentially observed in inner membrane spanning domains.

### Direct interaction between PorX and PorY

**Figure 2:**
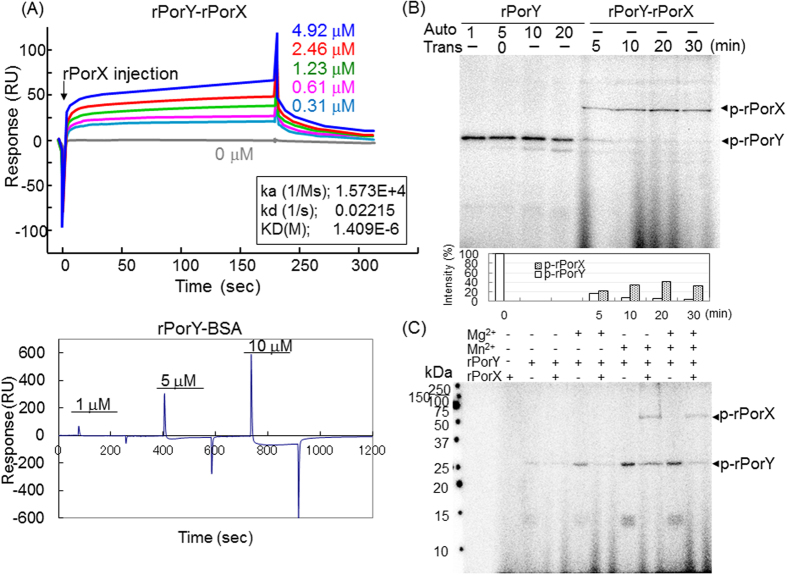
Interaction between PorX and PorY. (**A**) Affinity assays between rPorY and rPorX by surface plasmon resonance (SPR) analysis. rPorX (0.31–4.92 μM) were injected into the sensor chip immobilized by rPorY. BSA exhibited a negative interaction with rPorY. (**B**) *In vitro* phosphorylation of rPorY and rPorX. For autophosphorylation assay, rPorY (1 μg) was incubated with [^32^P-γ]ATP for 1, 5, 10, and 20 min at 0 °C. For transphosphorylation assay, rPorX (5 μg) and excess cold ATP (1 mM) were added to aliquots of rPorY 5 min after start of autophosphorylation and further incubated at 37 °C for indicated periods. The relative intensities of the radiolabelled protein bands were indicated in the graph after normalization. (**C**) The effects of compounds on the autophosphorylation of rPorY and the transfer of a phosphoryl group from rPorY to rPorX were examined. Phosphorylation assays were performed in the presence or absence of 10 mM MgCl_2_ and 1 mM MnCl_2_.

To investigate the direct interaction between PorX and PorY, the binding activity of recombinant PorX (rPorX) and recombinant PorY (rPorY) was determined by a real-time surface plasmon resonance (SPR) assay using BIAcore apparatus (Fig. 2A). Full-length rPorX was dose-dependently and stably bound to rPorY immobilized on CM5 sensor chips under physiological conditions [10 mM Hepes (pH 7.5) and 0.15 M NaCl]. The dissociation constant (K_D_) was calculated as 1.409 × 10^−6^ M with 1.573 × 10^−4^ (Ms)^−1^ of ka and 2.215 × 10^−2^ s^−1^ of kd. In contrast, binding of bovine serum albumin (BSA) to rPorY was not detected under the same conditions.

To examine the interaction specificity between rPorX and rPorY, we determined whether rPorX could bind to another HK of *P. gingivalis*, FimS (Supplemental Fig. 1). FimS/FimR is one of the TCSs in *P. gingivalis* which regulates the expression of a fimbrial component, FimA^24^,^25^. rPorX showed no significant interaction with recombinant FimS (rFimS), although recombinant FimR (rFimR) was bound to rFimS in a dose-dependent manner. The results clearly indicate that rPorX binds specifically to rPorY.

### Autophosphorylation of PorY and its *in vitro* phosphorelay to PorX

Signal transduction through TCS typically involves the autophosphorylation of HK at a histidine residue followed by transfer of the HK histidine residue phosphoryl group to an aspartate residue of RR. The resulting phosphorylated RR further modulates the transcription of effector genes^26^. Because the SPR assay demonstrated a direct interaction between PorX and PorY, we next examined the autophosphorylation of PorY and the transfer of a phosphoryl group to PorX using [γ-^32^P]ATP. As shown in Fig. 2B, rPorY with a molecular mass of 26 kDa was radiolabelled within 1 min, indicating rapid autophosphorylation. Additional incubation of phosphorylated rPorY with rPorX resulted in the transfer of the radiolabelled signal to a 55-kDa protein band corresponding to rPorX. The radioactivity of the 55-kDa rPorX band was increased in a time-dependent manner as revealed by the densitometrical analysis. These results strongly indicate that PorY and PorX act as an HK and RR of a cognate TCS, respectively, and regulate the secretion of virulence factors through the T9SS in *P. gingivalis*.

### Mn^2+^ requirement in the activation of PorXY TCS

A divalent cation around the phosphorylation site is considered necessary to add or remove phosphoryl groups in RRs. Mg^2+^ is preferred for many RRs, but other cations such as Mn^2+^ can be required for the reaction in some RRs. Therefore, we examined the effects of Mg^2+^ and Mn^2+^ on the phosphorylation of rPorY and rPorX. rPorY was autophosphorylated in the presence of either Mg^2+^ or Mn^2+^, whereas rPorY-mediated phosphorylation of rPorX required Mn^2+^, but not Mg^2+^ (Fig. 2C).

### *In vivo* phosphorylation of PorX by PorY

Phosphorylation of a protein leads to a change in its isoelectric point (p*I*), revealed by a p*I* shift in two-dimensional (2D) Western blotting. As shown in Supplemental Fig. 2a, three protein spots reacting with anti-PorX were detected in a 2D blot of *P. gingivalis* ATCC 33277 (wild-type) whole cell lysate. In the *porY* mutant, the spot with the higher p*I* value (solid arrowhead) was intensified compared with the wild type, whereas the intensities of the middle and lower p*I* spots (arrow and open arrowhead) were decreased. To confirm that the p*I* shift was caused by a phosphorylation state, whole cell lysate from the *porY* mutant was treated with lambda protein phosphatase (PP), or the direct phosphor donor acetyl phosphate (AcP), and then subjected to 2D-Western blotting (Supplemental Fig. 2b). The spot with the higher p*I* value was increased by PP treatment (solid arrowheads), while the spots with lower p*I* values and a higher molecular mass were increased by AcP treatment (open arrowheads). These results were not inconsistent with the *in vitro* result that PorX was phosphorylated by PorY.

### Involvement of SigP in the transcription of T9SS components

**Figure 3:**
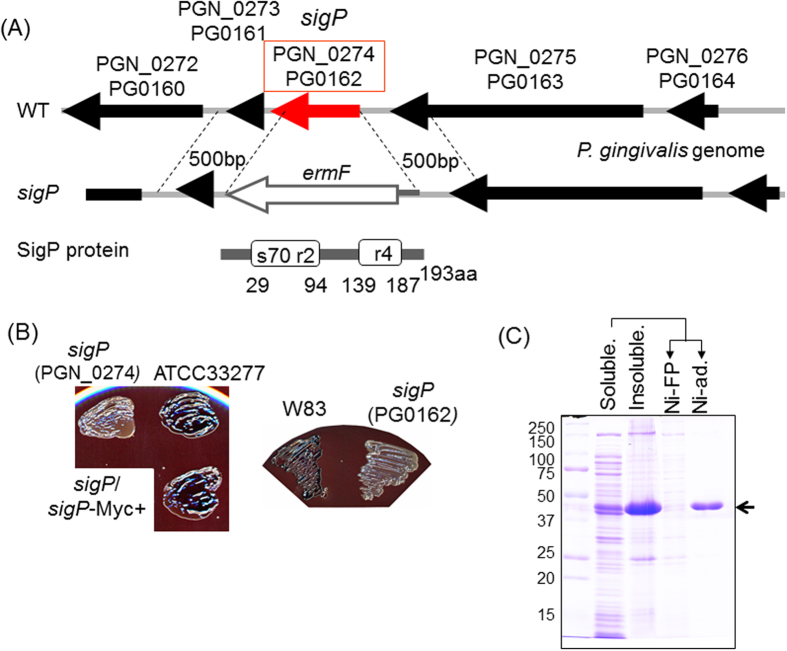
Construction of *sigP* deletion mutants and the complement strain and their colonial pigmentation. (**A**) Gene and protein structure of *P. gingivalis sigP*. The deletion mutants were generated by double recombination of the 500-bp targeted genes and the introduction of *ermF*. (**B**) Colony pigmentation in *P. gingivalis sigP* deletion mutants and the complement strain. (**C**) Purification of the recombinant SigP containing N-terminal thioredoxin- and C-terminal His_6_-tags. *E. coli* cells expressing the rSigP protein were lysed by sonication followed by centrifugation to separate a soluble fraction (Soluble) and an insoluble fraction (Insoluble). The soluble cell extracts were applied to nickel-nitrilotriacetic acid agarose to separate a free passed fraction (Ni-FP) and an adsorbed fraction (Ni-ad.).

The 193-amino acid protein PGN_0274 in strain ATCC 33277 (PG0162 in strain W83) encoded by *sigP* is predicted to be an ECF sigma factor with sigma-70 subdomains, the highly conserved region 2, and a helix-turn-helix r4 (Fig. 3A). Recently, mutants deficient in *sigP* have been reported to show decreased gingipain activity^19^ and increased biofilm formation^23^. To confirm the relationship between *sigP* and colonial pigmentation associated with gingipain activity, we constructed *sigP* deletion mutants (KDP391 and KDP392) from strains ATCC 33277 and W83, respectively, and a complement strain, KDP393. The *sigP* mutants exhibited non-pigmented colonies, while the complement strain (KDP393) and wild type strains (ATCC 33277 and W83) showed vigorous black pigmentation (Fig. 3).

**Figure 4:**
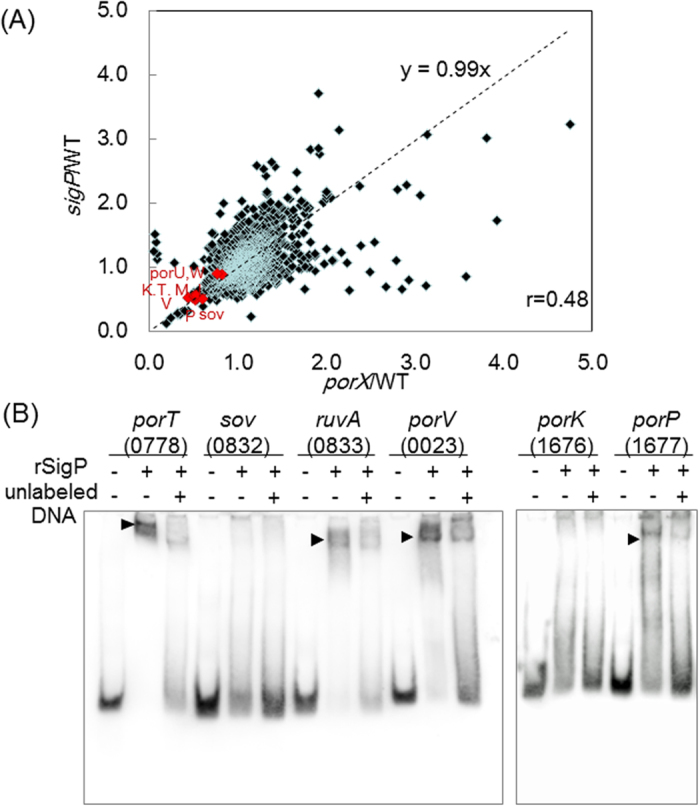
Involvement of SigP in the transcription of T9SS components. (**A**) Correlation in gene expression between *porX* mutant (KDP363) and *sigP* mutant (KDP314). Gene expression was measured by the custom tiling microarrays spanning the whole genome of *P. gingivalis* ATCC 33277. The expression level for each coding sequence was normalized with the constant from the 16S rRNA gene and represented as a ratio to that from wild type. Experiments were performed three times with independently prepared labelled cDNAs. The genes encoding T9SS components are represented in red. (**B**) EMSA assay of the promoter regions of T9SS component genes by rSigP. Probes corresponding to the possible promoter regions were generated by PCR and labelled with digoxigenin. Binding specificity was tested by competition with 100-fold excess of the appropriate unlabelled probe. PGN numbers for genes are indicated in parenthesis.

To determine which genes are influenced by *sigP*, microarray analysis of a *sigP* mutant (KDP314)^23^ using a custom tiling DNA array chip was performed. The 40 genes most down-regulated by the disruption of *sigP* are compared with those from the *porX* deletion mutant in Table 1. The expression of *porP*, *sov*, *porR*, *porK*, *porT*, *porM*, and *porL*, which are involved in the T9SS, decreased to less than 60% in the *sigP* mutant, which was the same as in the *porX* mutant. The decreased transcription of these genes was also confirmed by quantitative real time RT-PCR (Supplemental Fig. 3). Furthermore, 26 of the 40 down-regulated genes were common to *sigP* and *porX* mutants. A gene expression profile from the custom tiling DNA array also revealed the similarity in gene expression between *sigP* and *porX* mutants (correlation coefficient, r = 0.48) (Fig. 4A). These results indicate that the expression of a set of genes is regulated by both PorX and SigP.

### Binding of rSigP to the promoters of T9SS component genes

We next examined the ability of rSigP to bind to the promoter regions of genes listed in Table 1. Previous tiling array analysis revealed that *ruvA-sov* and *porP-porK-porL-porM-porN* regions produce polycistronic transcripts at least in part^11^, and that their expression was significantly downregulated in *porX* and *sigP* mutants (Supplemental Fig. 4). Since *ruvA* and *sov* seem to have no functional relationship; *ruvA* encodes a Holliday junction DNA helicase and *sov* encodes a T9SS component protein, and since Fjoh_3477 and FP2412, the orthologs of *porP* in *Flavobacterium johnsoniae* and *Flavobacterium psychrophilum* respectively, are separately located from *porK-porL-porM-porN* in their genomes, we determined whether rSigP binds to DNA regions 5′-adjacent to *sov* and *porK* in addition to *porT, ruvA, porV* and *porP*. The mobilities of DNA probes corresponding to the putative promoters of *porT* (encoding PGN_0788), *ruvA* (encoding PGN_0833), *porV* (encoding PGN_0023), and *porP* (encoding PGN_1677) shifted more slowly in the presence of rSigP (Fig. 4B). Addition of an excess amount (100-fold) of unlabelled probes significantly reduced the mobility shifts, confirming the specificity of the binding. On the other hand, rSigP showed no binding to the DNA regions 5′-adjacent to *sov* and *porK*.

DNA sequences of the probes for *porV*, *porT*, *ruvA* and *porP* in electrophoretic mobility shift assays (EMSA) were analyzed by the computational Motif Discovery scan, which examines DNA sequence motifs representing protein-DNA interaction sites (Supplemental Fig. 5). The consensus promoter motif, 5′-C(T)AAA(G/C/T)A(T)A(C)T(A/G)A-3′, was suggested to be a SigP target promoter motif. The predicted sequence was also found in the upstream regions of PGN_1353, PGN_1534, PGN_1639, PGN_1047 and PGN_0460, which were the coding sequences markedly downregulated in the *sigP* deletion mutant.

### Lack of SigP in the *porX* deletion mutant

**Figure 5:**
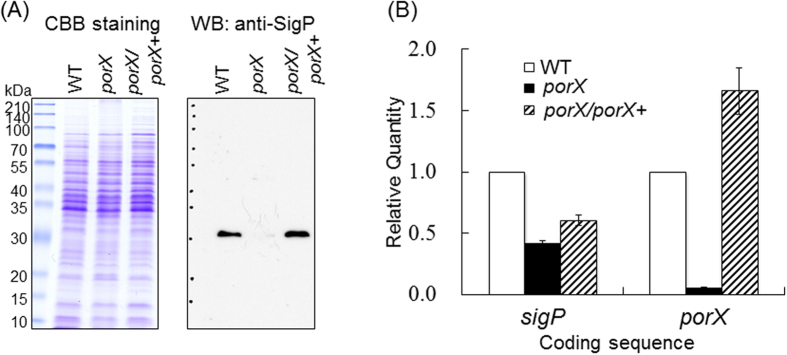
Expression of SigP in the *porX* mutant. (**A**) Western blot analysis of SigP in ATCC 33277 (WT), a *porX*-deficient mutant (KDP363), and the *porX/porX*^+^ complement strain (KDP372). Whole cell lysates from each strain corresponding to 5 μl of bacterial culture at OD_595_ 1.0 were applied to SDS-PAGE and transferred onto a PVDF membrane, followed by immunodetection with anti-SigP antibody. (**B**) Quantitative real time RT-PCR experiments with total RNA extracted from exponentially growing bacterial cells (OD_595_ 0.6) were performed.

To demonstrate the relationship between SigP and PorX, we examined the presence of SigP in the *porX* deletion mutant by Western blot analysis using an anti-SigP antibody (Fig. 5A). An approximately 30-kDa protein band corresponding to SigP disappeared in the *porX* deletion mutant, whereas the band was clearly detected in the wild-type and complemented *porX*/*porX*^+^ strains. To determine whether the lack of SigP in the *porX* mutant was caused at the transcriptional level, quantitative real time RT-PCR was performed (Fig. 5B). *sigP* mRNA levels in the *porX* mutant and the complemented *porX*/*porX*^+^ strain were shown to be decreased to 41% and 60% of that in wild type, respectively.

### Direct interaction between PorX and SigP

**Figure 6:**
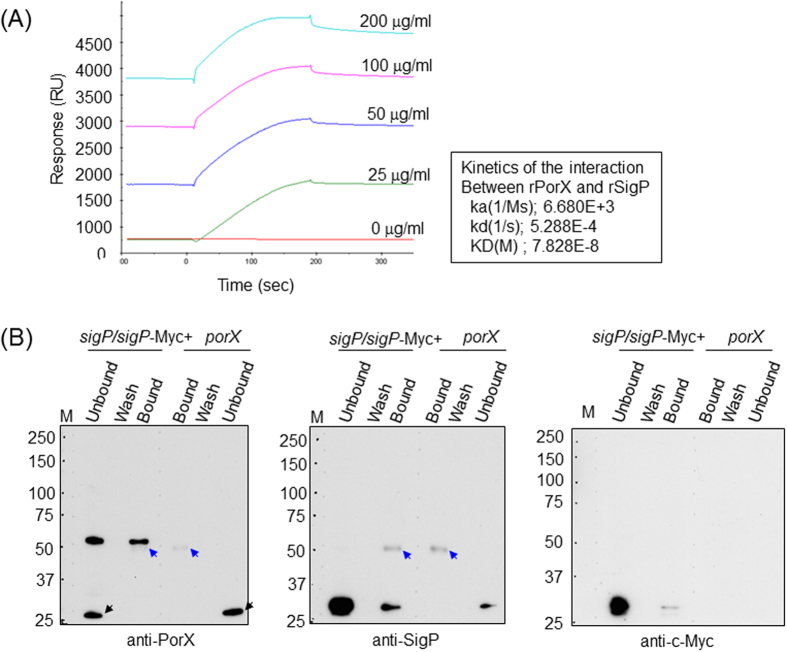
Interaction between PorX and SigP. (**A**) Affinity assays between rPorX and rSigP by SPR analysis. rSigP (0–200 μg/ml) were injected into the sensor chip immobilized by rPorX. (**B**) Co-immunoprecipitation of SigP by anti-PorX antibody. *P. gingivalis* lysates from *sigP/sigP*-Myc + complement and *porX*-deficient strains were incubated with anti-PorX antibody conjugated with Protein G sepharose. The unbound, wash, bound fractions were analyzed by Western blotting with anti-PorX (left), anti-SigP (middle), and anti-c-Myc (right) antibodies. Blue arrows (left and middle) and black arrows (left) indicate the heavy chain derived from antibodies and a nonspecific protein, respectively.

To investigate the direct interaction of PorX and SigP, binding of rSigP to rPorX were determined by SPR analysis with BIAcore (Fig. 6A). rSigP bound to rPorX immobilized on a CM5 sensor chip at the dissociation constant (K_D_) of 7.828 × 10^−8^ M. To examine the *in vivo* interaction between PorX and SigP, co-immunoprecipitation on *P. gingivalis* lysates was performed. Using anti-PorX antibody-conjugated protein G sepharose beads, SigP protein was co-precipitated with PorX in a *sigP/sigP*-Myc + complement strain as revealed by immunoblot detection with anti-SigP antibody, whereas SigP protein band was not detected in the precipitated fraction from a *porX* mutant (Fig. 6B middle panel). The co-immunoprecipitation of SigP was confirmed by use of anti-c-Myc antibody (Fig. 6B right panel). These results indicated that PorX and SigP physically interacted with each other in *P. gingivalis* cells.

## Discussion

We previously demonstrated that the T9SS was involved in the secretion of virulence factors responsible for colony pigmentation, haemagglutination, adherence, the modification of bacterial surface proteins, and the degradation and/or activation of host proteins. T9SS substrate proteins are translocated across the inner membrane via Sec machinery, and then secreted through the outer membrane by the T9SS in association with their C-terminal domains^27^. Thus, the T9SS machinery seems to be crucial for the determination of bacterial pathogenicity. In the present study, we identified a regulatory cascade system for the T9SS of *P. gingivalis*, namely, the TCS composed of PorX and PorY and its downstream ECF sigma factor, SigP.

We found a direct interaction, including a phosphorelay, between PorX and PorY in this study. The interaction was characterized by an affinity (K_D_) of 1.4 μM (Fig. 2), which compares with known average affinities between other HKs and RRs of 1.2 μM (EvgS/EvgA), 0.9–1.3 μM (CheA/CheY), and 0.12 μM (PhoQ/PhoP)^28^,^29^,^30^, demonstrating that PorX and PorY are a cognate pair of a TCS, and an RR and HK, respectively although *porX* and *porY* are “orphan” genes.

A divalent cation is considered necessary to add or remove phosphoryl groups in RRs. PhoPQ TCS is known to be important for virulence regulation in *Salmonella typhimurium*, in which PhoP acts as an RR that is activated under low Mg^2+^ concentrations^31^. The chemotaxis protein Y (CheY) from *Escherichia coli* is an RR that regulates chemotactic flagellar rotation, which also requires the presence of Mg^2+ ^32. Cyanobacterial phytochrome Cph1, a light regulated HK, and *Chlamydia trachomatis* TCS, CtcB-CtcC, were previously reported to be highly activated in the presence of Mn^2+ ^33,34. In this study, we found that Mg^2+^ or Mn^2+^ was required for autophosphorylation of PorY and that transphosphorylation from PorY to PorX required Mn^2+^, but not Mg^2+^ (Fig. 2C). Requirement of the divalent cation for the reactions suggests that phosphorylation of the PorXY TCS is similar to that of other known TCSs.

Besides the PorXY TCS, four TCS have been characterized in *P. gingivalis* to date: (1) the FimRS system comprising FimR (PGN_0903) and FimS (PGN_0904), which are involved in fimbriation^24^; (2) the predicted hybrid HK with an RR domain, GppX (PGN_1768), which is associated with black pigmentation and the secretion of gingipains^35^; (3) the stress response RR, RprY (PGN_1186), whose cognate kinase has not yet been identified^36^; and (4) the PGN_0752-PGN_0753 system, which participates in bacterial haemin acquisition^37^. Wild-type gingipains were previously shown to mainly associate with cells, although some are released into the culture supernatant; this differs from the gingipains of *gppX*-deficient mutants, which are typically found in the supernatant^35^. Therefore, GppX is thought to be involved in the regulation of biosynthesis of an anionic lipopolysaccharide, A-LPS, that anchors gingipains to the cell surface.

RRs are typically composed of two domains, a phosphor–receiver domain and an effector domain. Functions of the latter domain can be classified as DNA binding (63%), enzymatic activity (13%), protein binding (3%), RNA binding (1%), and no functional domain (17%)^38^. PorX lacks a typical DNA binding motif such as a helix-turn-helix, but contains a PglZ domain at its C terminus (Fig. 1). In fact, EMSA revealed that rPorX did not bind the upstream regions of genes encoding T9SS components which are downregulated in the *porX*-deficient mutant (data not shown).

A similar set of genes including T9SS components were found to be controlled by both PorX and SigP in the present study (Fig. 4, Table 1), while the *sigP* mutant phenotype was similar to those of *porX* and *porY* mutants^19^. EMSA revealed that rSigP could bind to predicted promoter regions of *porT* (PGN_0788), *ruvA* (PGN_0833), *porV* (PGN_0023), and *porP* (PGN_1677), but not to the upstream regions of *sov* (PGN_0832) and *porK* (PGN_1676). These results indicate that genes encoding T9SS components are directly regulated by the SigP sigma factor.

We found that SigP protein levels were barely detectable in the *porX* mutant, and that *sigP* mRNA expression was 40% that of wild type. In contrast, the amount of SigP in the *sigP*/*sigP*^+^ complemented strain was similar to wild type, although *sigP* mRNA levels in the complemented strain were 60% that of wild type (Fig. 5). This suggests that SigP sigma factor is unstable in the absence of PorX.

The direct interaction between PorX and SigP *in vitro* and *in vivo* were demonstrated by SPR analysis and co-immunoprecipitaion, respectively, in this study (Fig. 6). Interaction between RRs and sigma factors was previously reported in *E. coli* RssB adaptor, a member of the RR family and an RpoS sigma factor^39^. In this case, a complex of RpoS and RssB is rapidly degraded by the ATP-dependent protease ClpXP. Under stress conditions, the small anti-adaptor proteins IraP, IraM, and IraD, which form under different stress conditions, bind to the RssB adaptor and block RpoS degradation, resulting in the activation of RpoS-dependent genes. The RssB C terminus does not contain a DNA-binding domain and has similarities to PP2C Ser/Thr phosphatases^40^,^41^. Although the interaction between PorX and SigP in *P. gingivalis* is similar to that between RssB and RpoS in *E. coli*, the consequence of the PorX-SigP interaction appears to differ from that of RssB–RpoS because the SigP sigma factor is present and active in the presence of PorX and lacking in its absence.

The regulatory system of T9SS in *P. gingivalis* could be a novel therapeutic target for periodontal diseases caused by this bacterium because the machinery contributes to the secretion of various virulence factors, including gingipains. Functional T9SSs have been experimentally demonstrated in *Cytophaga hutchinsonii*^42^, *Flavobacterium johnsoniae*^43^, *Tannerella forsythia*^44^,^45^, and *Capnocytophaga ochracea*^46^, in addition to *P. gingivalis.* Moreover, genome analysis has revealed that many bacteria belonging to the phylum *Bacteroidetes* possess T9SS component and substrate genes^47^. An understanding of T9SS expression will help in the development of disease control of infections associated with T9SS-possessing bacteria.

## Methods

### Bacterial strains and culture conditions

Bacterial strains and plasmids used in this study are listed in Supplemental Table 1. *P. gingivalis* cells were grown anaerobically (80% N_2_, 10% CO_2_, 10% H_2_) in enriched brain heart infusion broth (Becton Dickinson, Franklin Lakes, NJ) and on enriched tryptic soy agar (Nissui, Tokyo, Japan) supplemented with 5 μg/ml haemin (Sigma, St. Louis, MO), and 0.5 μg/ml menadione (Sigma). For blood agar plates, defibrinated laked sheep blood was added to enriched tryptic soy agar at 5%^8^. Luria–Bertani (LB) broth (Sigma) and LB agar plates were used to grow *E. coli* strains. Antibiotics were added to the medium for the selection and maintenance of antibiotic-resistant strains at the following concentrations: ampicillin (100 μg/ml for *E. coli*), erythromycin (10 μg/ml for *P. gingivalis*).

### Construction of *P. gingivalis* deletion mutants

*P. gingivalis* deletion mutants (KDP391 and KDP392 from ATCC 33277 and W83, respectively) were generated by double recombination of the targeted genes and the introduction of erythromycin resistance genes, as previously described^11^. Primers used in this study are listed in Supplemental Table 2, and experimental details are described in Supplemental Text.

### Construction of complemented *sigP*-deficient mutant strain

The entire *sigP* containing its promoter was PCR-amplified from chromosomal DNA using primers N0274-U-F-K and N0274-D-R-X, digested with *Kpn*I and *Xho*I, and then inserted into the *Kpn*I–*Not*I site of the pTCB plasmid carrying Myc-and His_6_-tags, and the transcriptional terminator DNA region (pKD911). After mating of *E. coli* S17-1 containing pKD911 and the *P. gingivalis sigP* deletion mutant, an Em^r^ Tc^r^ transconjugant (KDP393) was obtained.

### Subcellular fractionation

Subcellular fractionation of *P. gingivalis* cells was performed as previously described^48^. Further details are given in Supplemental Text.

### Preparation of recombinant proteins

Full-length DNA (1,557 bp) of *porX* and the histidine kinase domain-coding 3′-region of *porY* (648 bp; nt 537–1185) were PCR-amplified from the chromosomal DNA of *P. gingivalis* ATCC33277 (Fig. 1). The *Nde*I–*Xho*I fragment including *porX* or the *Nde*I–*Xho*I fragment including *porY* were inserted into the expression vector pET28a(+) (Merk Novagen, Darmstadt, Germany).

DNA lacking a termination codon of *sigP* (579 bp) was PCR-amplified from the chromosomal DNA of *P. gingivalis* ATCC 33277 (Fig. 3). The *BamH*I–*Xho*I fragment including *sigP* was inserted into the expression vector pET32b(+) (Merk Novagen).

The pET28a(+) plasmids containing the N-terminal His_6_-tagged *porX* or the 3′-region of *porY*, and the pET32b(+) plasmid containing N-terminal thioredoxin- and C-terminal His_6_-tagged *sigP* were transformed into *E. coli* BL21(DE3), and gene expression was induced by the addition of isopropyl-β-D-thiogalactopyranoside to a final concentration of 1.0 mM, and growth was continued for 3 h. The recombinant PorX, PorY, and SigP were purified with nickel–nitrilotriacetic acid (Ni-NTA) agarose (Thermo Fisher, Waltham, MA). Details are given in Supplemental Text.

### Preparation of antisera

Antisera against PorX, PorY, and SigP protein were raised in rabbits using recombinant proteins. Full-length PorX and SigP, and the C-terminal region of PorY lacking transmembrane domains were expressed with tags of thioredoxin and His_6_ in the pET32b system, followed by purification with Ni-NTA agarose. Rabbit antibodies against PorX or SigP were obtained by binding to Protein A sepharose. Monoclonal anti-c-Myc antibody was purchased from Sigma.

### Immunoprecipitation

Antibody against PorX was chemically crosslinked to Protein G sepharose with 50 mM dimethyl pimelimidate (Sigma). *P. gingivalis porX*-deficient and *sigP/sigP*-Myc + complement strains were lysed in BugBuster Protein Extraction Reagent (Merk Novagen) and insoluble debris was removed by centrifugation at 27,000 × *g* for 30 min. The supernatants were incubated with anti-PorX antibody-conjugated protein G sepharose and washed 5 times with 50 mM Tris-HCl buffer containing 0.15 M NaCl and 0.05% NP-40, pH7.5. Associated proteins were eluted by boiling in a sample buffer.

### Gel electrophoresis and Western blot analysis

Western blots were performed to detect PorX, PorY and SigP in extracts of wild-type and mutant cells of *P. gingivalis*. A total of 10 μg protein, as determined by a BCA assay (Thermo Fisher), was loaded per lane and proteins were separated on 12% polyacrylamide gels and subjected to sodium dodecyl sulfate-polyacrylamide gel electrophoresis (SDS-PAGE). Proteins were transferred onto polyvinylidene difluoride (PVDF) membranes, then immunodetected with anti-PorX or anti-PorY antiserum, and anti-SigP or anti-c-Myc antibodies obtained in this study.

### Binding affinity analysis with BIAcore surface plasmon resonance

The binding affinity of rPorX to rPorY and rSigP to rPorX was investigated by SPR using a BIAcore X100 biosensor (GE Healthcare, Little Chalfont, UK). For the experiment, rPorY or rPorX proteins were immobilized onto a carboxylated dextran matrix sensor chip (CM5) resulting in an average bound density of 3000 response units. The series diluents of the recombinant rPorX were used as analytes in a running buffer [10 mM Hepes (pH 7.5) and 0.15 M NaCl] at a 30-μl/min flow rate. The association was recorded in the form of sensorgrams which were used to calculate association and dissociation constants. Following each cycle, the flowcell surfaces were regenerated with 30 μl of 0.1 M Tris-HCl (pH 8.0) containing 1 M NaCl at a 10-μl/min flow rate and re-equilibrated in the running buffer.

BiaEvalution software (version X100 from GE Healthcare) was used to determine the simultaneous dissociation (kd) and association (ka) rates by non-linear fitting assuming a 1:1 complex model. The equilibrium dissociation constants (K_D_) were then calculated as kd/ka. Binding data are summarized in Figs 2A and 6A from the average of three complete sets of data.

### *In vitro* phosphorylation and phosphorelay assays

For the autophosphorylation assay, rPorY was incubated in a kinase buffer [25 mM Tris-HCl, pH 7.5, 10 mM MgCl_2_, 1 mM MnCl_2_, 2 mM DTT, 5 mM β-glycerophosphate, 0.1 mM Na_3_Vo_4_ and 0.5 μCi of [γ-^32^P]ATP ] for 1, 5, 10, or 20 min at 0 °C. Phosphotransfer assays were conducted by allowing rPorY to autophosphorylation for 5 min, followed by the addition of purified rPorX with excess cold ATP (1mM). Reactions were stopped at indicated times. Samples were mixed with SDS loading buffer and subjected to SDS-PAGE for separation in 12% polyacrylamide gels. After electrophoresis, gels were dried and exposed to Imaging Plates (GE Healthcare).

### Tiling microarray analysis

Custom tiling microarrays spanning the whole genome of *P. gingivalis* ATCC33277 with 25-mer probes (each of which was eight-bases shifted on the genome sequence) were purchased from Affymetrix (Santa Clara, CA). The analytical procedure was described previously^11^. Details are given in Supplemental Text.

### Electrophoretic mobility shift assays (EMSA)

EMSAs were performed using the DIG Gel Shift Kit (Roche) according to the manufacturer’s instructions. Probes used in this assay were designed from the possible transcription starting sites in the tiling microarray, and PCR-amplified from *P. gingivalis* ATCC 33277 DNA using primers shown in Supplemental Table 2. Labelled probes (4 ng) were incubated with rSigP (1 μg) in 20 μl binding buffer for 30 min at room temperature. The reactions were run on 6% Novex DNA gel Retardation gels in 0.5 × TBE buffer (44.5 mM Tris, 44.5 mM boric acid, 1 mM EDTA, pH 8.0) at 4 °C. DNA–protein complexes were electrically transferred to Hybond-N^+^ membranes (GE Healthcare), and DIG-labelled reactions detected with chemiluminescence using an alkaline phosphatase-conjugated anti-DIG antibody (Roche) and CDP-star detection reagent (Roche).

### Quantitative real-time RT-PCR

Total RNA was transcribed into cDNA with SuperScript III first-strand synthesis system (Thermo Fisher). Quantitative real-time RT-PCR was performed with the Brilliant II Fast SYBR green QPCR master mix (Agilent Technologies, Santa Clara, CA) and Mx3005P real-time PCR system (Agilent Technologies) according to the manufacturer’s instructions. Primers for real-time analysis (Supplemental Table 2) were designed using Primer3 software (http://primer3.sourceforge.net/). Quantitative real-time PCR conditions were as follows: 1 cycle at 95 °C for 2 min, then 35 cycles of 95 °C for 5 s and 60 °C for 20 s. At each cycle, the accumulation of PCR products was detected by the reporter dye from the double-stranded DNA binding SYBR green. To confirm that a single PCR product was amplified, a dissociation curve was constructed in the range of 55–95 °C after the PCR. All data were analysed using Mx3005P software. The expression level of each targeted gene was normalized to that of 16S rRNA. All PCRs were carried out in triplicate. The efficiency of primer binding was determined by linear regression by plotting the cycle threshold (*C*_*T*_) value versus the log of the cDNA dilution. Relative quantification of the transcript was determined using the comparative *C*_*T*_ method (2^−ΔΔCT^) calibrated to 16S rRNA. Experiments were independently performed three times with comparable results.

## Additional Information

**How to cite this article**: Kadowaki, T. *et al.* A two-component system regulates gene expression of the type IX secretion component proteins via an ECF sigma factor. *Sci. Rep.* **6**, 23288; doi: 10.1038/srep23288 (2016).

## Supplementary Material

Supplementary Information

## Figures and Tables

**Table 1:**
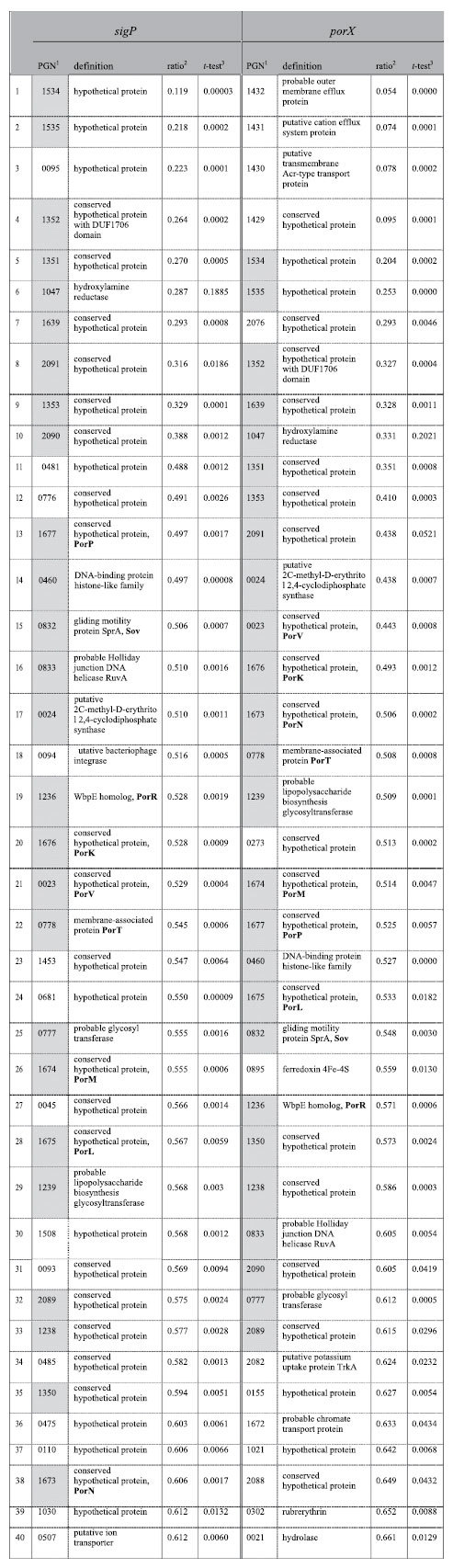
Genes down-regulated in *sigP*-and *porX*-deficient mutants.

^1^PGN coding sequences down-regulated in both mutants are highlighted. ^2^Values represent the ratio of the expression level in the *sigP* mutant (KDP314) or *porX* mutant (KDP363) to that in the wild type (ATCC 33277). ^3^Values represent two-sample assuming unequal variances according to Student’s *t*-test.
