# Supplementary material for: A two-component system regulates gene expression of the type IX secretion component proteins via an ECF sigma factor
Source: Sci Rep. 2016 Mar 21;6:23288. doi: 10.1038/srep23288 (PMC4800418; doi:10.1038/srep23288)
Supplement: Supplementary Information [file srep23288-s1.pdf]

Supplemental Information for:

A two-component system regulates gene expression of the type IX secretion component proteins via an ECF sigma factor

Tomoko Kadowaki[1,2], Hideharu Yukitake[1], Mariko Naito[1], Keiko Sato[1], Yuichiro Kikuchi[3], Yoshio Kondo[1,4], Mikio Shoji[1], & Koji Nakayama[1*]

[1]Division of Microbiology and Oral Infection, Department of Molecular Microbiology and Immunology, and [2]Division of Frontier Life Science, Department of Medical and Dental Sciences, Nagasaki University, Nagasaki 852-8588, Japan. [3]Department of Microbiology, Tokyo Dental College, Tokyo 101-0061, Japan. [4]Department of Pediatric Dentistry, Graduate School of Biomedical Sciences, Nagasaki University, Nagasaki 852-8588, Japan. *Correspondence and requests for materials should be addressed to K.N. (email: knak@nagasaki-u.ac.jp)

Supplemental Figure 1| Affinity assays between rPorX and rFimS by SPR analysis. rFimR and rFimS were expressed from pET28a(+) inserted by PCR-amplified *fimR* or *fimS* from the chromosomal DNA of *P. gingivalis* ATCC 33277. (**a**) rFimR (1–10 μM) was injected into the sensor chip immobilized by rFimS. (**b**) rPorX (0.27–2.73 μM) was injected into the sensor chip immobilized by rFimS. (**c**) BSA exhibited a negative interaction with rFimS.

Supplemental Figure 2| Comparative 2D-western blot analysis of PorX in the cell lysates of *P. gingivalis*. (**a**) 2D-PAGE with IPG strips covering isoelectric points 4–7 was performed using whole cell extracts from wild-type (WT) and a *porY*-deficient mutant (KDP364). PorX was detected by immunoblotting with anti-PorX antiserum. Proteins were stained with Coomassie Brilliant Blue. The right lower insets in each Western blotting show the magnifications of the immunoreacting spots. The relative densities of the spots were indicated in the insets. (**b**) Cell lysate from a *porY*-deficient mutant was treated with phosphatase inhibitors (PPI; 5 mM orthovanadate, 5 mM NaF, 10 mM imidazole, and 20 mM sodium tartrate), lambda protein phosphatase (PP), or acetyl phosphate (AcP), and then applied to 2D-Western blot analysis. Experiments

performed three times with independently prepared cell lysates, and the representative is shown.

Supplemental Figure 3| Gene expression of T9SS components and gingipains in *P. gingivalis* wild-type (WT) and *sigP* mutant. *P. gingivalis* ATCC 33277, KDP391 were cultivated to the optimal density (OD_{595}) 0.6. Quantitative real-time RT-PCR was performed using previously described primers [1]. PGN numbers for genes are indicated in parenthesis.

1. Sato, K. *et al.* A protein secretion system linked to bacteroidete gliding motility and pathogenesis. *Proc. Natl. Acad. Sci. U. S. A.* **107**, 276-281, doi:10.1073/pnas.0912010107 (2010).

Supplemental Figure 4| Tiling microarray analysis of *porX* and *sigP* mutants.

Tiling microarray analysis in the vicinities of *porU-porV* (a), *porT* (b), *ruvA-sov* (c) and *porP-porN* (d) were shown.

Supplemental Figure 5| Prediction of targeted promoter motif by SigP. The DNA sequences of the probes for *porV, porT, ruvA* and *porP* in EMSA (Figure 4) were analyzed by the computational method, Motif Discovery scan (MDscan), that examines

the DNA sequence motifs representing the protein-DNA interaction sites (http://ai.stanford.edu/~xsliu/MDscan/). The predicted sequence was also found in the upstream regions of CDSs affected by *sigP* deletion.

Supplemental Figure 1

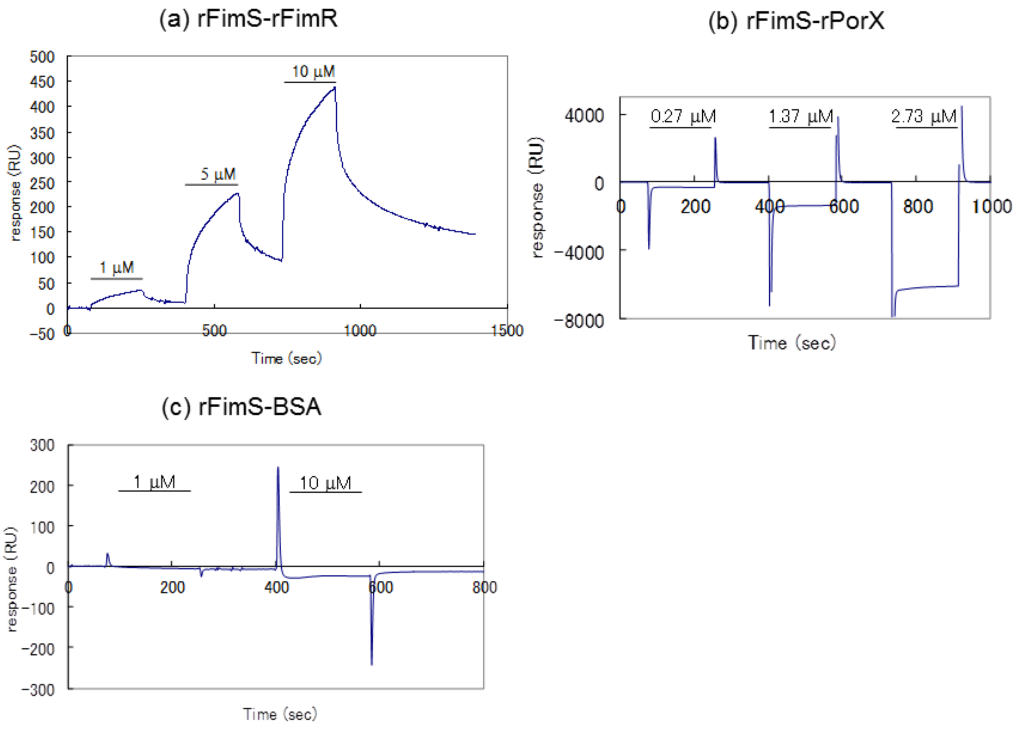

Supplemental Figure 2

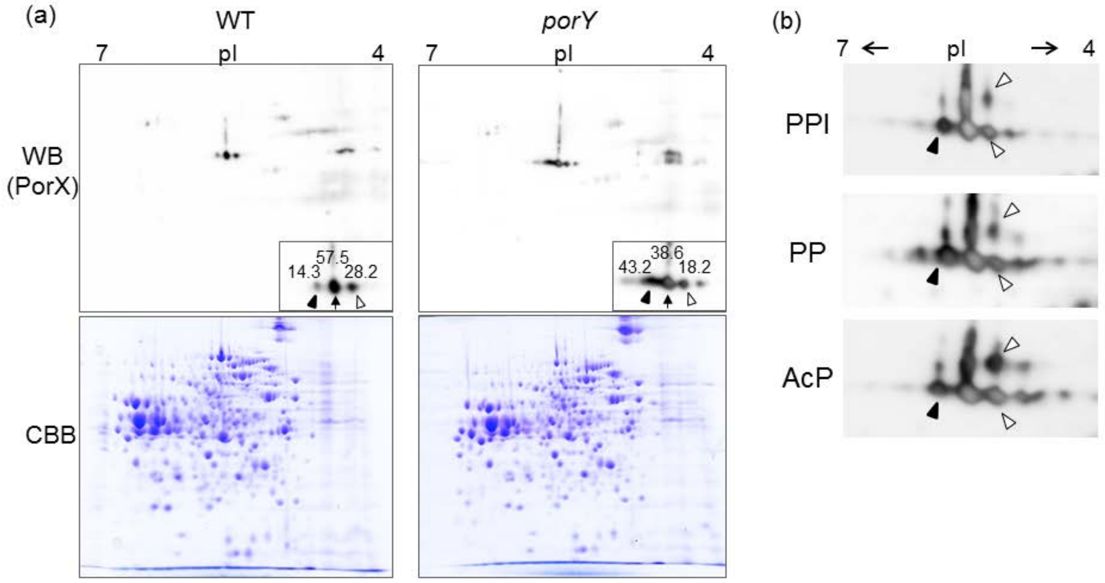

Supplemental Figure 3

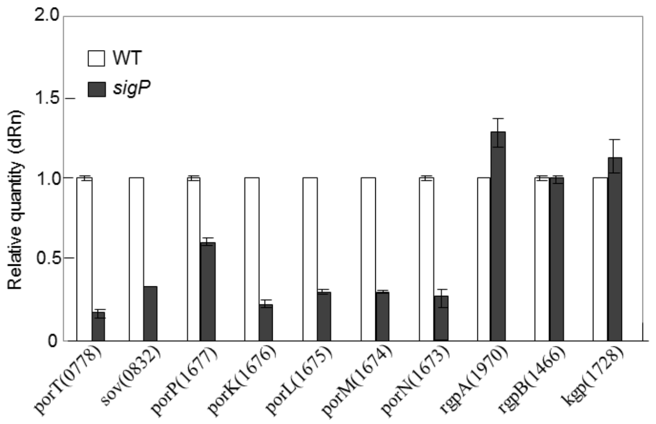

Supplemental Figure 4

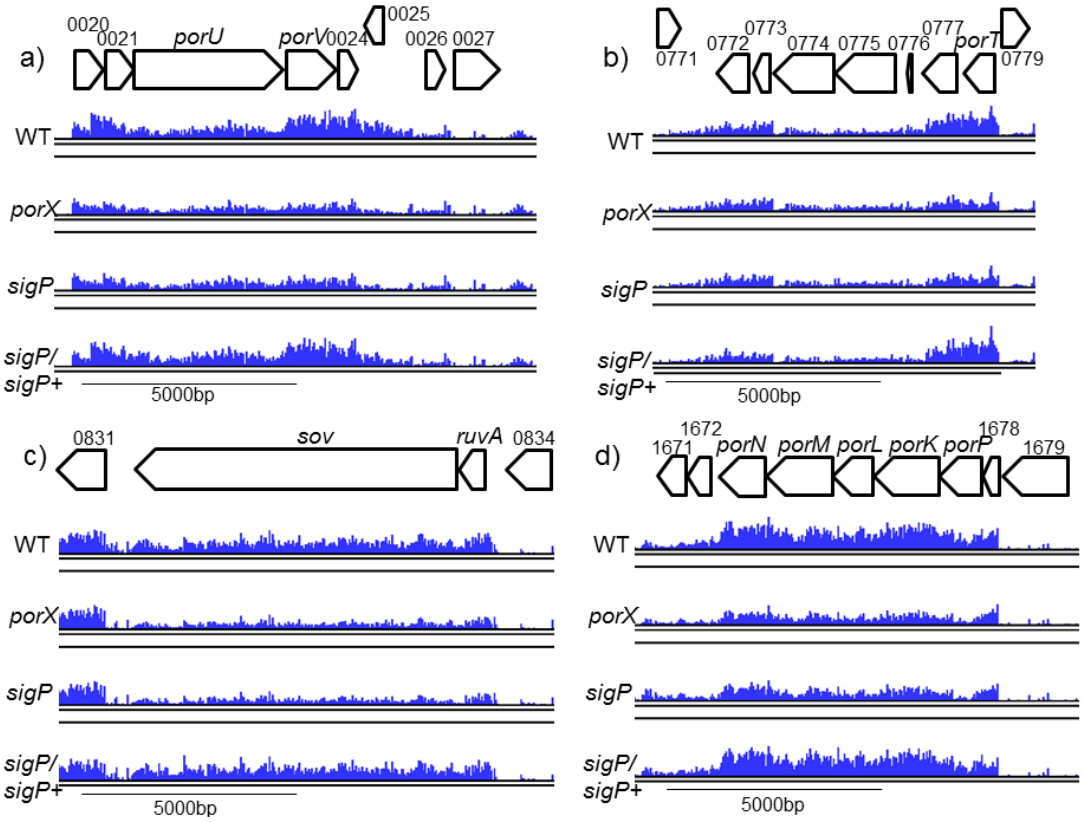

Supplemental Figure 5

Gene	CDS	Start	Site	Sequence
porV	PGN0023	29901	−103	CCTCCATGGC CAAGAAAA TGATCGTGGT
			− 28	TAGACCCCGA TAAGAATA CAAAACAAAA
			− 20	GATAAGAATA CAAAACAA AACGAACGAA
			− 15	GAATACAAAA CAAAACGA ACGAAATATG
porT	PGN0778	847377	−113	GTAGGTTATG CAAATCTA CGAACGAGAT
			− 75	CCGATTCGCG CAAAAATA CAATATCTTT
ruvA	PGN0833	924187	−123	TAGTAATAAA CAACACTA TGGTGGCTTT
porP	PGN1677	1873093	− 45	CCCGGATGTG CAATAATA GCACTCGGGG
−	PGN1353	1510110	−264	CTTAACCTCG CAAAGATA GCCGTTTTCT
−	PGN1534	1874117	−189	ACGCTCGGTA CAAACATA ACATTTTTCC
−	PGN1639	1834485	−249	TAGTCATGTA CAAACAGA TACAGGCAGC
−	PGN1047	1165206	−105	CGCCGCATAG CAAGAGTA CCCGGTGTCG
−	PGN0460	502076	−216	ATGATATGCT CAAAAATG TCTGTCAGAT

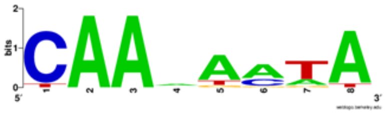

Supplemental Table 1| Bacterial strains and plasmids used in this study.

Strain or plasmid	Description	Reference or source
E. coli strain		
BL21(DE3)	host strain for expression vectors	*Nippongene*
S17-1	RP4-2-Tc::Mu *aph*::Tn7 *recA*, Sm^r	1
P. gingivalis strain		
ATCC 33277	wild type	
W83	wild type	
KDP314	*sigP*::*ermF ermAM*, Em^r	2
KDP363	*ΔporX*::*ermF*, Em^r	3
KDP364	*ΔporY*::*ermF*, Em^r	3
KDP372	KDP363 *fimA::*[*porX*$^+$ *tetQ*], Em^r Tc^r	3
KDP391	*ΔsigP*::*ermF*, Em^r, ATCC 33277 derivative	this study
KDP392	*ΔsigP*::*ermF*, Em^r, W83 derivative	this study
KDP393	KDP391/pKD911, Em^rTc^r	this study
Plasmid		
pTCB	Ap^r Tc^r, *E. coli*-*P. gingivalis* shuttle plasmid	4
pKD911	Ap^rTc^r, pTCB-promoter-*sigP*$^+$-*myc*-terminator	this study

1. Simon R, Priefer U, & Puhler A (1983) A broad host range mobilization system for in vivo genetic engineering: transposon mutagenesis in Gram negative bacteria. *Bio/Technology* **2**, 784-791 (1983).

2. Onozawa, S. *et al.* Role of extracytoplasmic function sigma factors in biofilm formation of *Porphyromonas gingivalis*. *BMC Oral Health* **15**, 4, doi:10.1186/1472-6831-15-4 (2015).

3. Sato, K. *et al.* A protein secretion system linked to bacteroidete gliding motility and pathogenesis. *Proc. Natl. Acad. Sci. U. S. A.* **107**, 276-281, doi:10.1073/pnas.0912010107 (2010).
4. Nagano K, *et al.* Characterization of RagA and RagB in *Porphyromonas gingivalis*: study using gene-deletion mutants. *J. Med. Microbiol.* **56**, 1536-1548 (2007).

Supplemental Table 2| Primers used in this study.

Primer	sequence	Description
Expression of recombinant proteins		
N1019F-N-28	catatggaaaaaaacatgagaccgtatacc	*Nde*I-*porX* (N1019)
N1019R-X-28	ctcgagttacttgggttgcatcgtaattac	*Xho*I-*porX* (N1019)
N1019F-B-32	ggatccgatggaaaaaaacatgagaccgta	*Bam*HI- *porX* (N1019)
N1019R-X-32	ctcgagcttgggttgcatcgtaattacggg	*Xho*I-*porX* (N1019)
N2001F-N-28	catatggagcgcagatccgaacagaatcgt	*Nde*I-*porX* (N2001)
N2001R-X-28	ctcgagttatggcagatcggtctgtttcgg	*Xho*I-*porX* (N2001)
N2001F-E-32	gatatcgcagaatcgtgtatggaacggact	*Eco*RV-*porY* (N2001)
N2001R-X-32	ctcgagtggcagatcggtctgtttcggcag	*Xho*I-*porX* (N2001)
N0903F	gctagcatgattagtatcgtactcgtggat	*Nhe*I-*fimR* (N0903)
N0903R	ctcgagttactattgccaatccactaatccgc	*Xho*I-*fimR* (N0903)
N0904F	catatgaggagatacagacaaaataagaga	*Nde*I-*fimS*(N0904)
N0904R	ctcgagttattacgctgaggcaaggacaaaag	*Xho*I-*fimS*(N0904)
N0274F	ggatccgatgagcagtttccacaagctgac	*Bam*HI-N0274
N0274R	ctcgagagccgacatgcccatcattttgcg	*Xho*I-N0274
Mutation		
N0274-U-F	gcggccgcaactactgctactgtctcggac	*Not*I-N0274-U

N0274-U-R	ggatccaggagaatcctctttatttattgt	*Bam*HI-N0274-U
N0274-D-F	ggatccagagattgcagatgctaccggagt	*Bam*HI-N0274-D
N0274-D-R	ggtacctttatatgtaatgctgcaaagtta	*Kpn*I-N0274-D
Complementation		
N0274-U-F-K	ggtaccatggtgaaccgtaccgagcattgc	*Kpn*I-N0274-U
N0274-D-R-X	ctcgagagccgacatgcccatcattttgcg	*Xho*I-N0274-D
qRT-PCR		
REALpgn0274F	gagccaggttgcagtctttc	
REALpgn0274R	gctcatgtcctcccagtagc	
REALpgn1019F	gatcggggacagaagtacca	
REALpgn1019R	attcgggtaggcgaagaagt	
pg16SrF1	cttgacttcagtggcggca	
pg16SrR1	agggaagacggttttcacca	
EMSA		
N0023pro-F	cagacggcctccatggccaagaaaatgatc	N0023 promoter
N0023pro-R	atttcgttcgttttgttttgtattcttatc	N0023 promoter
N0778pro-F	ataattattgttgtgaggtaggttatgcaa	N0788 promoter
N0778pro-R	tcaacgccttatgcagcaaagatattgtat	N0788 promoter
N0832pro-F	ggctatacgcaaagcagtcaagtccattct	N0832 promoter
N0832pro-R	gagaaaaggttataacattcgtaatgctcg	N0832 promoter
N0833pro-F	ttagtgactgaaatgcgattgccctgttac	N0833 promoter
N0833pro-R	caccatagtgttgtttattactataggaaa	N0833 promoter
N1676pro-F	ttctttcccattgagcaatacgaagaatag	N1676 promoter

N1676pro-R	acattatttatatatctgcttcgctgctct	N1676 promoter
N1677pro-F	gcaaagaggattttttcttcaacgaagaag	N1677 promoter
N1677pro-R	gactgatgccacggtggtaaaacgaagccc	N1677 promoter

Supplemental Text 1| Construction of *P. gingivalis* deletion mutants

P. gingivalis deletion mutants were generated by double recombination of the targeted genes and the introduction of erythromycin resistance genes. DNA regions upstream and downstream of *sigP* were PCR amplified from the chromosomal DNA of *P. gingivalis* ATCC 33277 using pairs of primers, N0274-U-F and N0274-U-R, N0274-D-F and N0274-D-R. The amplified DNA upstream of *sigP* was double digested with *Not*I and *Bam*HI, while DNA downstream of the gene was digested with *Kpn*I and *Bam*HI. Both digested products were ligated together with pBluescript II SK(−), which had been digested with *Not*I and *Kpn*I. The 1.1-kb *Bam*HI *ermF* DNA fragment was inserted into the *Bam*HI site of the resulting plasmids to yield plasmids for mutagenesis. These plasmids were digested with *Not*I and *Kpn*I and introduced into *P. gingivalis* ATCC 33277 or W83 by transformation. In the case of double-deletion mutants, the inserted *ermF* on one gene was replaced with *tetQ* after digestion with *Bam*HI and *Bgl*II, and introduced into the first gene-mutated *P. gingivalis*.

Supplemental Text 2| Subcellular fractionation.

P. gingivalis cells from a 400 ml culture were harvested by centrifugation at 10,000 × *g* for 30 min at 4°C, washed twice and resuspended in 40 ml of buffer A [10 mM Hepes–NaOH, pH 7.4, containing 0.15 M NaCl, 0.1 mM N^{α}-*p*-tosyl-L-lysine chloromethyl ketone (TLCK), 0.1 mM leupeptin, and 0.2 mM phenylmethylsulfonyl fluoride (PMSF)]. The cells were disrupted by passing twice through a French pressure cell at 100 MPa. The remaining intact bacterial cells were removed by centrifugation at 2,400 × *g* for 10 min, and the supernatant was subjected to ultracentrifugation at 100,000 × *g* for 60 min. The pellets were gently agitated with buffer A containing 1% Triton X-100 and 20 mM $MgCl_2$ for 30 min at 20°C to solubilize the inner membrane. The outer membrane fraction was recovered as a precipitate by ultracentrifugation at 100,000 × *g* for 60 min at 4°C. The supernatant was obtained as the inner membrane fraction.

Supplemental Text 3| Preparation of recombinant proteins.

Full-length DNA (1,557 bp) of *porX* was PCR-amplified from the chromosomal DNA of *P. gingivalis* ATCC 33277 (Figure 1). The forward primer (N1019F), attached by an *Nde*I site, and the reverse primer (N1019R), attached by an *Xho*I site, were designed to

preserve the reading frame through the N-terminal His_6-tag. The amplified DNA was extracted from an agarose gel and cloned into the pGEM-T Easy Vector (Promega, Madison, WI.). The *Nde*I–*Xho*I fragment of the plasmid was inserted into the expression vector pET28a(+).

The histidine kinase domain-coding 3′-region of *porY* (648 bp; nt 537–1185) was PCR-amplified from the chromosomal DNA of *P. gingivalis* ATCC 33277 (Figure 1). The forward primer (N2001F), attached by an *Nde*I site, and the reverse primer (N2001R), attached by an *Xho*I site, were designed to preserve the reading frame through the N-terminal His_6-tag. The amplified DNA was extracted as before, cloned into the pGEM-T Easy Vector, and the *Nde*I–*Xho*I fragment inserted into pET28a(+).

DNA lacking a termination codon of *sigP* (579 bp) was PCR-amplified from the chromosomal DNA of *P. gingivalis* ATCC 33277 (Figure 3). The forward primer (N0274F), attached by a *Bam*HI site, and the reverse primer (N0274R), attached by an *Xho*I site, were designed to preserve the reading frame through the N-terminal thioredoxin- and C-terminal His_6-tags. The amplified DNA was extracted as before, cloned into the pGEM-T Easy Vector, and the *Bam*HI–*Xho*I fragment inserted into the expression vector pET32b(+).

The pET28a(+) plasmids containing the N-terminal His_6-tagged *porX* or the 3′-region of *porY*, and the pET32b(+) plasmid containing C-terminal His_6-tagged *sigP* were transformed into *E. coli* BL21(DE3), and transformants were selected on LB plates containing ampicillin (100 μg/ml). *E. coli* BL21(DE3) harbouring *porX/Y* or *sigP*-containing plasmids were cultivated in 500 mL of LB broth at 30°C until an OD_{595} of 0.6 was reached. Gene expression was induced by the addition of isopropyl-β-D-thiogalactopyranoside to a final concentration of 1.0 mM, and growth was continued for 3 h. Cells were harvested by centrifugation at 6000 × *g* for 10 min.

Bacterial cells were resuspended in 50 mM Tris-HCl buffer (pH 8.0) containing 0.1 M NaCl and 1 mM PMSF (WAKO), and washed twice with the same buffer. The cell suspension was lysed by sonication followed by centrifugation for 15 min at 27 000 × *g*. The resulting supernatants (soluble cell extracts) were loaded onto a pre-equilibrated nickel–nitrilotriacetic acid (Ni-NTA) agarose (Invitrogen). The resin was washed with 50 mM Tris-HCl buffer (pH 8.0) containing 0.1 M NaCl, 1 mM PMSF, and 10 mM imidazole, and eluted by ascending the imidazole concentration to 300 mM. The eluted samples were immediately concentrated with Centriprep centrifugal filters (Merk Millipore), and dialyzed against 50 mM Tris-HCl buffer (pH 7.5) containing 0.15 M KCl.

Supplemental Text 4| Tiling microarray analysis.

Custom tiling microarrays spanning the whole genome of *P. gingivalis* ATCC 33277 with 25-mer probes (each of which was eight-bases shifted on the genome sequence) were purchased from Affymetrix. The analytical procedure was described previously [11]. In brief, antisense biotinylated cDNA was prepared from 10 μg of total RNA with a reverse transcriptase (Superscript II; Invitrogen) and random hexamer primers were used to produce DNA complementary to the RNA. cDNA products were then fragmented by DNase I and labelled at the 3′ termini with terminal transferase and biotinylated Gene Chip DNA labelling reagent (Affymetrix). The fragmented and labelled cDNA was hybridized to the Gene Chip at 45°C for 16 h. Signal intensities were quantified with the Gene Chip Operating Software (Affymetrix), and further data analyses were performed with the Genomic Analysis Program (Insilico Molecular Cloning Array edition, In Silico Biology). The normalization constant from the 16S rRNA gene was used to calculate the calibrated ratio for each coding sequence. Experiments were performed three times with independently prepared labelled cDNAs.
